# Towards a Novel Biocontrol Strategy: High Performance of Optimised Cell Wall‐Degrading Enzymes Secreted by *Escovopsis primorosea* LBM 277

**DOI:** 10.1111/1758-2229.70271

**Published:** 2026-03-18

**Authors:** Marcela Paola Barengo, Natalia Soledad Amerio, Gustavo Ángel Bich, Pedro Darío Zapata, María Lorena Castrillo

**Affiliations:** ^1^ Molecular Biotechnology Laboratory Institute of Biotechnology Misiones “Dra. María Ebe Reca”, Faculty of Exact, Chemical and Natural Sciences, National University of Misiones Garupá Argentina; ^2^ CONICET (National Scientific and Technical Research Council) Buenos Aires Argentina

**Keywords:** chitinases, leaf‐cutter ants, mycoparasitism, proteases, submerged fermentation, β‐1,3‐glucanases

## Abstract

Fungi of the genus *Escovopsis* are specialised mycoparasites of the mutualistic fungus *Leucoagaricus gongylophorus*, cultivated by leaf‐cutter ants. Harnessing this natural antagonism represents a promising and environmentally friendly strategy for the indirect biological control of these agroforestry pests. This study optimised and characterised the production of cell wall‐degrading enzymes (CWDEs), key effectors in the degradation of the host, by the *Escovopsis primorosea* LBM 277 strain. Using submerged fermentation and Box–Behnken response surface methodology (RSM‐BBD), we identified optimal culture conditions, including the concentrations of carbon and nitrogen sources, initial pH and inoculum size. Under optimised conditions, protease, β‐1,3‐glucanase and chitinase activities increased by 3.5‐, 6.7‐ and 6.8‐fold, respectively. The enzymes remained active for at least 30 days at room temperature and in the pH range 4–6, resembling the microenvironment of ant fungal gardens. Zymographic analysis revealed one isoform of protease, one of β‐1,3‐glucanase and two chitinase isoenzymes. This is the first report combining RSM‐based optimization with biochemical profiling of CWDEs in *Escovopsis*. These findings highlight the need for further functional assays to validate the role of these enzymes in the ant–symbiont fungus–mycoparasite interaction.

## Graphical abstract

*Escovopsis primorosea* LBM 277 efficiently produced proteases, β‐1,3‐glucanases and chitinases under submerged fermentation using *Leucoagaricus gongylophorus* cell walls and Mandels complex. RSM‐BBD optimization significantly increased enzyme activity. The enzymatic cocktail remained stable for 30 days at room temperature and pH 4–6, aligning with the conditions found in leaf‐cutter ant nests. These findings highlight the strain's potential as a biotechnological tool for indirect biological control through targeted enzymatic degradation of the fungal symbiont cultivated by leaf‐cutter ants.

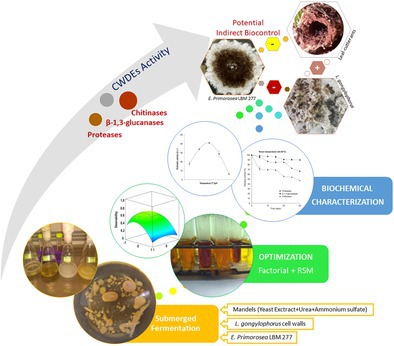

## 1 Introduction

The evolution of agroforestry production systems has created the need to implement biological control methods within the integrated pest management (IPM) approach. These methods aim to mitigate the risks associated with the indiscriminate use of pesticides, safeguard human health and the environment and foster sustainable agroforestry development (Gelot et al. 2024; Zhou et al. 2024; Fenibo and Matambo 2025).

The study of various fungal species and their bioactive compounds has gained significant interest in this context due to their potential as biological control agents. These fungi can act as natural antagonists to reduce the damage caused by harmful organisms, positioning them as a promising source for developing sustainable strategies in pest management (Ram et al. 2018; Amerio et al. 2020; de Sousa et al. 2025).

Leaf‐cutter ants (*Atta*, *Acromyrmex* and *Amoimyrmex*) are among the most significant pests in the subtropical and tropical regions of America due to their extensive defoliation capacity (Della Lucia and Amaral 2020; Müller et al. 2024; Masiulionis and Samuels 2025). The most extensively studied strategy for their control involves using entomopathogenic fungi, with *Beauveria bassiana* and *Metarhizium anisopliae* being the most prominent (Sullivan et al. 2022; Folgarait and Goffré 2023; Leal et al. 2024). However, developing more effective biological control agents requires exploring a broader range of microorganisms and understanding their mechanisms of action (Teodoro et al. 2023; Villavicencio‐Vásquez et al. 2025).

Leaf‐cutter ants cultivate gardens of the fungus *Leucoagaricus gongylophorus* (Basidiomycota: Agaricales), their principal food source. In turn, fungi of the genus *Escovopsis* (Ascomycota: Hypocreales) are specialised mycoparasites of *L. gongylophorus*, as they depend exclusively on its tissues for development (Currie et al. 2003; Haifig 2014; Elliot et al. 2025). As a result, *Escovopsis* can significantly reduce the biomass of the symbiotic fungus, indirectly affecting the survival of ant colonies. Due to this impact, *Escovopsis* is emerging as a potential indirect biological control agent, offering a new perspective for the sustainable management of these pests (Queiroz et al. 2024).

Mycoparasitic fungi penetrate the host mycelium by secreting extracellular cell wall‐degrading enzymes (CWDEs) that hydrolyze its structural polymers (Lorito et al. 1994). These enzymes—primarily proteases, chitinases and β‐1,3‐glucanases—act synergistically on chitin, β‐1,3‐glucans and proteins, playing a key role in fungal antagonism (Kuhn et al. 2012; Sahgal 2022; Brazhnikova et al. 2025). Consequently, the application of these enzymes in biotechnology introduces a ‘green technology’ approach, with the potential to develop more sustainable and efficient biopesticides for biological control (Ferreira and de Freitas Soares 2023; Mejía et al. 2024). For their production, submerged fermentation (SmF) is the most widely used technology, as it allows precise control of the physicochemical parameters of the culture medium and facilitates the recovery of enzyme products, among other advantages of the system (Gong et al. 2023; Nazir et al. 2024).

The optimization of culture medium composition and fermentation conditions is crucial to maximising enzyme production. Carbon and nitrogen sources, initial pH and inoculum concentration significantly affect enzyme production (Dukariya and Kumar 2021; He et al. 2023; Amerio et al. 2025). However, no defined culture medium has been established for optimal production of microbial CWDEs, since each microorganism has specific requirements for its enzyme secretion (Shi et al. 2024; Liu et al. 2025).

Although *Escovopsis* has been recognised as a specialised mycoparasite of *L. gongylophorus*, most research has focused on three aspects: (i) the pathogenicity mechanisms based on physical contact between both fungi (Haifig 2014; Marfetán et al. 2015), (ii) the production of secondary metabolites with antifungal activity (Dhodary et al. 2018; Heine et al. 2018; Batey et al. 2020) and (iii) the response of *Escovopsis* to volatile and non‐volatile organic compounds generated by *Leucoagaricus* (Masiulionis and Pagnocca 2020; Berasategui et al. 2024; de Oliveira et al. 2024). However, studies on the production and characterisation of extracellular enzymes in *Escovopsis* remain scarce (Inglis and Kawchuk 2002; de Man et al. 2016; Barengo et al. 2020; Gotting et al. 2022), highlighting the need to investigate their enzymatic capabilities and potential as a tool in biocontrol strategies.

Therefore, this study aimed to optimise the production of CWDEs by *Escovopsis primorosea* LBM 277 using SmF. Subsequently, the enzymes present in the optimised supernatant were biochemically characterised to determine their optimal reaction parameters, stability under various pH and temperature conditions and isoenzyme profiles.

## 2 Materials and Methods

### 2.1 Microorganisms

This study utilised two fungal strains sourced from the collection of the Molecular Biotechnology Laboratory at the Misiones Biotechnology Institute (InBioMis), of the National University of Misiones (UNaM), Argentina.

*E. primorosea* LBM 277 was previously selected by our research group as a promising mycoparasite due to its significant antagonistic activity against *L. gongylophorus* strains (data not shown). The strain was cultivated in Petri dishes containing modified Czapek yeast agar (mCYA) with the following composition: KNO_3_ (0.03 M), K_2_HPO_4_ (5.74 × 10^−3^ M), KCl (6.7 × 10^−3^ M), MgSO_4_·7H_2_O (2.02 × 10^−3^ M), FeSO_4_·7H_2_O (3.7 × 10^−4^ M), ZnSO_4_·7H_2_O (8.7 × 10^−6^ M), CuSO_4_·5H_2_O (2 × 10^−5^ M), yeast extract (5 g L^−1^), dextrose (30 g L^−1^) and agar (20 g L^−1^). The cultures were incubated at 28°C ± 1°C in the dark for 5–7 days.

*L. gongylophorus* LBM265 was selected as the inducer because earlier assays comparing different fungal cell wall sources indicated a significantly higher CWDEs production when using this strain's cell walls (data not shown). This strain was grown in Petri dishes containing 3.9% w/v potato dextrose agar (PDA) medium with 0.02% w/v of chloramphenicol and was incubated at 28°C ± 1°C for 14 days.

### 2.2 Carbon and Nitrogen Sources

The cell walls of *L. gongylophorus* LBM265 were utilised as a carbon source. Agar discs coated with the fungus mycelium were inoculated in 2% (w/v) malt extract (ME) medium and incubated at 28°C ± 1°C for 15 days in darkness. The mycelial cell walls were then extracted following the procedure described by Cortes et al. (1998). Purification was conducted using 0.85% (w/v) NaCl for 2 h, then subjected to boiling in 2% sodium dodecyl sulphate (SDS) for 5 min to solubilise internal tissues. The obtained material was sequentially washed with a chloroform: methanol (1:1, v/v) solution, then with acetone, air‐dried and finally ground in a mortar.

The nitrogen sources were adapted from Mandels medium (Mandels and Reese 1957), whose basal components include KH_2_PO_4_ (2 g L^−1^), CaCl_2_·2H_2_O (0.4 g L^−1^), MgSO_4_·4H_2_O (0.3 g L^−1^), FeSO_4_·7H_2_O (0.005 g L^−1^), MnSO_4_·4H_2_O (0.0016 g L^−1^), ZnSO_4_·7H_2_O (0.0014 g L^−1^) and CoCl_2_·6H_2_O (0.02 g L^−1^). The nitrogenous components consist of urea (0.3 g L^−1^), yeast extract (0.25 g L^−1^) and ammonium sulphate (1.4 g L^−1^). In this study, the basal medium composition was maintained while modifications were made to the nitrogen sources.

### 2.3 Culture Conditions in SmF: Inoculum, Incubation and Sampling

The assays were carried out under SmF conditions. Erlenmeyer flasks of 250 mL containing 75 mL of the culture medium were sterilised and then inoculated with 3 mL of a spore suspension of 1 × 10^7^ spores mL^−1^ concentration. The cultures were incubated at 28°C ± 1°C in darkness, shaking at 100 rpm for 16 days. Periodically, 3 mL samples of supernatant were taken under sterile conditions. As this fungus forms compact mycelial pellets under these conditions, the cell‐free supernatant was used without further purification. The response of the assays was quantified by determining the activity of CWDEs present in these crude supernatants.

### 2.4 Spectrophotometric Determination of Enzymatic Activity

#### 2.4.1 Protease Activity

Protease activity was determined using a modified version of the method described by Charney and Tomarelli (1947), adapted by Barengo et al. (2020). The chromogenic substrate, 0.5% (w/v) azocasein, was dissolved in 0.2 M Tris–HCl buffer (pH 7.4). The reaction mixture, consisting of 300 μL azocasein and 300 μL enzyme supernatant, was incubated at 37°C for 50 min. A volume of 600 μL of 10% (w/v) trichloroacetic acid was added to terminate the reaction. The mixture was centrifuged at 7000 rpm for 5 min and the resulting supernatant was neutralised with 1 M NaOH. Absorbance was measured at 440 nm using a Shimadzu UV‐Vos 1900 spectrophotometer. Enzyme activity was defined in units equivalent to the activity of 1 mg of papain (1 U = 1 mg of papain), based on a standard curve prepared with commercial papain (Sigma‐Aldrich) using azocasein as substrate (Kole et al. 1988; Barengo et al. 2020).

#### 2.4.2 β‐1,3‐Glucanase Activity

β‐1,3‐glucanase activity was assessed following a modified protocol based on the method of Masih and Paul (2002). The substrate consisted of 1% (w/v) laminarin in 0.2 M acetate buffer (pH 5). A reaction mixture containing 250 μL of substrate and 250 μL of enzyme supernatant was incubated at 50°C for 40 min. The reaction was stopped by adding 500 μL of 3,5‐dinitrosalicylic acid (DNS) reagent and boiling for 10 min. After cooling, 4 mL of distilled water was added and absorbance was recorded at 540 nm. One unit (U) of β‐1,3‐glucanase activity was defined as the amount of enzyme required to release 1 μmol of glucose per minute under the assay conditions.

#### 2.4.3 Chitinase Activity

Chitinase activity was measured using a colorimetric method based on Kim et al. (2003), with modifications. The substrate consisted of 0.5% (v/v) colloidal chitin prepared in 0.05 M acetate buffer at pH 4.8. A volume of 300 μL substrate was mixed with 300 μL of enzyme supernatant and incubated at 37°C for 60 min with agitation at 90 rpm. The reaction was stopped with 600 μL of DNS reagent (Miller 1959), boiled for 10 min and centrifuged at 7000 rpm for 5 min. Absorbance was measured at 540 nm. One unit (U) of chitinase activity was defined as the amount of enzyme capable of releasing 1 μmol of *N*‐acetylglucosamine (NAG) per minute under the assay conditions.

### 2.5 Optimisation of Nutritional Conditions for Enhanced CWDEs Production

#### 2.5.1 Optimisation of Carbon and Nitrogen Sources

Determining the optimum carbon and nitrogen source concentrations involved stepwise optimization, adjusting one factor at a time. Parameters that had a statistically significant effect on enzyme production were identified and incorporated into subsequent experiments.

The carbon source was optimised using a four‐level factorial design. The effect of four concentrations of *L. gongylophorus* cell walls on the enzymatic activity of *E. primorosea* LBM 277 was evaluated. The assayed concentrations were 0.13, 0.36, 0.81 and 1.25 g L^−1^. All assays were performed in duplicate.

The optimization of nitrogen sources was carried out using the response surface methodology (RSM) generated with Statgraphics Centurion XVI (StatPoint Inc., USA). A Box–Behnken design (BBD) was selected due to its efficiency in identifying optimal conditions with fewer experimental points than other designs.

For this step, the basal components of Mandels medium were used, with modifications in nitrogen composition. The effects of three nitrogen sources—urea, yeast extract and ammonium sulphate—were assessed at three concentration levels (1, 0 and −1). To facilitate data interpretation, actual concentrations were converted into standardised units, where 1 represented the highest concentration and −1 the lowest. Seventeen assays were conducted, including a central point in quintuplicate. The central point, positioned at the 0 level for all factors, ensured a more uniform variance estimation across the design space. The coded values for low, middle and high levels of each variable are detailed in Table 1A.

**TABLE 1:** Coded factor levels for RSM‐BBD of three variables to optimise (A) nitrogen sources: urea, yeast extract, ammonium sulphate and (B) initial pH, inoculum concentration and urea levels.

Factor		Level		
		−1	0	+1
*A*	Urea (g L^−1^)	0.2	1.1	2
	Yeast extract (g L^−1^)			
	Ammonium sulphate (g L^−1^)			
*B*	pH	4	5.5	7
	Inoculum (spores mL^−1^)	1 × 10^6^	1 × 10^7^	2 × 10^7^
	Urea (g L^−1^)	0	0.1	0.2

#### 2.5.2 Optimisation of Initial pH, Inoculum Concentration and Urea Levels

To further enhance the CWDE activity of the *E. primorosea* LBM 277 strain, the following variables were optimised: urea concentration, initial pH of the medium and inoculum concentration. Optimization was conducted using the RSM‐BBD methodology, evaluating three levels (1, 0 and −1) for each factor. Seventeen assays were performed, including a central point in quintuplicate. The tested factors and their corresponding levels are detailed in Table 1B. Each culture medium incorporates the previously optimised variables.

#### 2.5.3 Statistical Analysis

The factorial design data were analysed using the InfoStat software version 2018 (InfoStat Group, Argentina). An analysis of variance (ANOVA) was conducted to determine the significant effects of carbon source concentration on enzymatic activity and differences between means were assessed using the Tukey test with a 95% confidence level.

In RSM‐BBD designs, data analysis was performed using Statgraphics Centurion XVI (StatPoint Inc., USA). An ANOVA within the RSM framework was conducted at a 95% confidence level. Model Quality in explaining experimental data was assessed through a lack‐of‐fit test and the coefficient of determination (*R* ^2^). Additionally, Pareto charts were used to identify the most significant factors. Three‐dimensional response surface plots were generated to visualise the relationship between enzyme activity and the levels of the tested variables. Optimal values for each variable were determined through multiple‐response optimization analysis.

### 2.6 Validation of the Optimised Experimental Conditions

A quintuple assay was performed using the optimal values for each variable to validate the optimal conditions predicted for each factor in the multiple‐response optimization analyses. Finally, the experimentally obtained enzymatic activity values were compared with the estimated values to assess the accuracy of the optimization.

### 2.7 Biochemical Characterisation of CWDEs Produced by *E. primorosea* LBM 277 in the Optimised Supernatants

#### 2.7.1 Effect of Temperature and pH on CWDEs Activity

Protease activity was assessed across a temperature range of 25°C–95°C and a pH range of 5–9. β‐1,3‐Glucanase activity was tested at 25°C–70°C and pH 3–7, while chitinase activity was measured at 25°C–65°C and pH 3–7. All other reaction conditions were maintained constant and the assays were run in two replicates.

#### 2.7.2 Thermal and pH‐Stability of CWDEs

The enzymatic supernatants were incubated at different temperatures and pH to assess enzyme stability post‐fermentation. Residual activity was measured and expressed as a percentage, with the initial enzyme activity (time zero) set as 100%.

Thermal stability was evaluated by incubating the supernatants at room temperature (24°C–28°C) and under refrigeration (5°C) for 30 days, with samples collected every 5 days. Additionally, stability was assessed at each enzyme's optimal reaction temperature, with samples taken every 5 min.

For pH stability, supernatants were incubated at values near the optimal reaction pH of each enzyme at room temperature. Stability was monitored over 30 days, with samples collected every 5 days.

The data for the effect of temperature and pH on CWDEs and enzymatic stability were processed using InfoStat version 2018 (InfoStat Group, Argentina). To analyse the influence of the variables on the enzymatic activity and stability, an ANOVA and a difference between means test were performed using the Tukey test, with a confidence level of 95.0%.

#### 2.7.3 Detection of Isoenzymes With CWDE Activity in Non‐Denaturing Electrophoresis in Polyacrylamide Gels

The presence of isoenzymes with CWDE activity (proteases, β‐1,3‐glucanases and chitinases) was detected through zymogram analysis using non‐denaturing electrophoresis in 7.5% (w/v) polyacrylamide gels (ND‐PAGE) (Laemmli 1970; Murugesan et al. 2007). Enzyme supernatants were clarified with a 0.1% (v/v) Tween 80 in a 2:1 ratio and 25 μL of each sample was loaded per lane. Electrophoresis was conducted at a constant voltage of 120 V, followed by incubation for 120 min in 1.5 M Tris‐Glycine buffer (pH 8.3).

##### 2.7.3.1 Proteases Zymography

ND‐PAGE was conducted using 0.3% (w/v) skimmed milk incorporated as a substrate in the gel to detect protease isoenzymes. After the electrophoretic run, the gel was incubated in 0.2 M Tris–HCl buffer (pH 7.4) for 15 min in a refrigerator, followed by 2 h at room temperature and then for 1 h at 37°C. The gel was stained for 1 h with a solution containing 0.25% (w/v) Coomassie blue, 30% (v/v) methanol and 10% (v/v) acetic acid. Finally, washes were performed with a solution of methanol 30% (v/v) and acetic acid 10% (v/v) until clear bands were visible on the blue background.

##### 2.7.3.2 β‐1,3‐Glucanases Zymography

For β‐1,3‐glucanase isoenzymes detection, ND‐PAGE was carried out with 0.1% (w/v) laminarin as substrate. Following the electrophoretic run, the gel was incubated in 0.2 M acetate buffer (pH 5) for 40 min at 50°C. The gel was stained for 15 min with gentle agitation in 0.1% (w/v) Congo red. Afterward, washes were performed with a 1 M NaCl solution until the bands were visible.

##### 2.7.3.3 Chitinases Zymography

The presence of chitinase isoenzymes was determined through ND‐PAGE, containing 0.1% (v/v) colloidal chitin incorporated as substrate in the gel. After performing the electrophoretic run, the gel was incubated in 0.05 M acetate buffer, pH 4.8, for 1 h at 45°C. The gel was stained for 15 min with gentle agitation in Congo red 0.1% (w/v). Finally, washes were performed with a 1 M NaCl solution until the bands were visible on the gel.

## 3 Results

### 3.1 Optimisation of Nutritional Conditions for Enhanced CWDEs Production

#### 3.1.1 Carbon and Nitrogen Sources

First, the determination coefficients obtained from the ANOVA (*R* ^2^ = 0.80 for protease, 0.95 for β‐1,3‐glucanase and 0.89 for chitinase) showed that the selected carbon source levels explained most of the observed variability, validating the reliability of the factorial design. The evaluation of the concentrations of *L. gongylophorus* LBM265 cell walls revealed increased activity for all three CWDEs when the culture medium contained 0.36 g L^−1^ of fungal cell walls.

**FIGURE 1:**
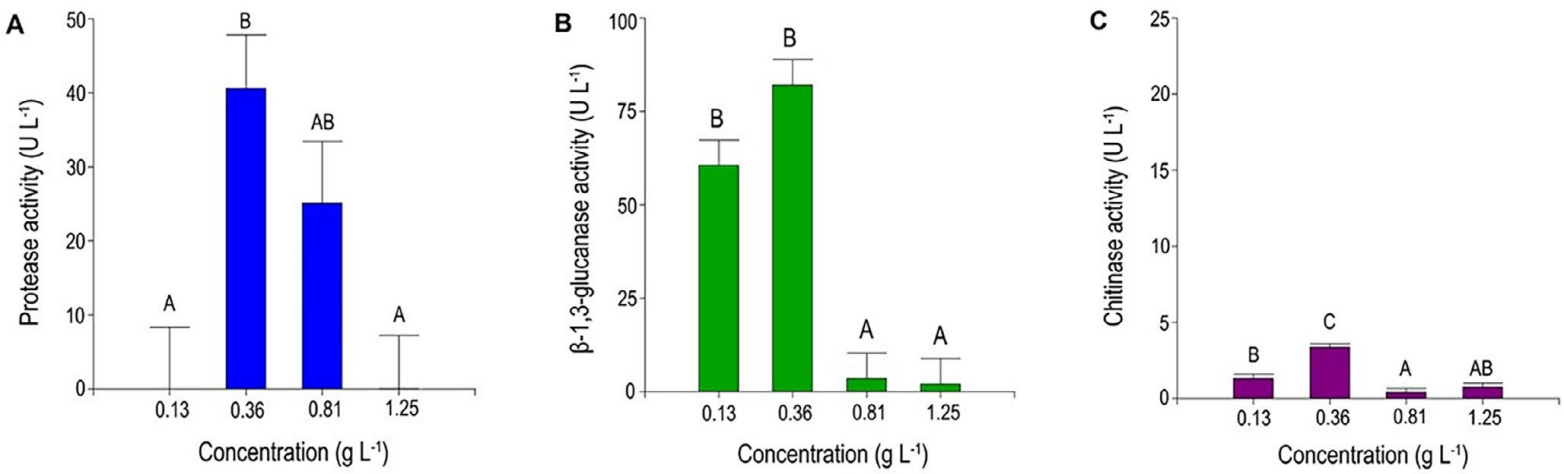
Effect of different concentrations of *Leucoagaricus gongylophorus* cell walls as a carbon source on: (A) protease activity, (B) β‐1,3‐glucanase activity and (C) chitinase activity of the *Escovopsis primorosea* LBM 277 strain. Statistical analysis was performed using Tukey's test with a 95% confidence interval. Different letters indicate significant differences (*p* < 0.05).

For proteases, the enzymatic activity at the optimal concentration showed statistically significant differences with the extreme levels of the design (*p* < 0.05) (Figure 1A). Regarding β‐1,3‐glucanase activity, significant differences were observed between the optimal carbon source concentration and the higher levels of the design (*p* < 05) (Figure 1B). Finally, chitinase activity at the optimal concentration was significantly higher than at all other tested levels (Figure 1C).

In the RSM ANOVA of the three concentrations for each nitrogen component of the Mandels medium (urea, yeast extract and ammonium sulphate), the *R* ^2^ value explained 88.04% of the variability in the results for protease activity. The lack‐of‐fit test was not significant (*p* > 0.05), indicating that the selected model was appropriate for describing the observed data.

**FIGURE 2:**
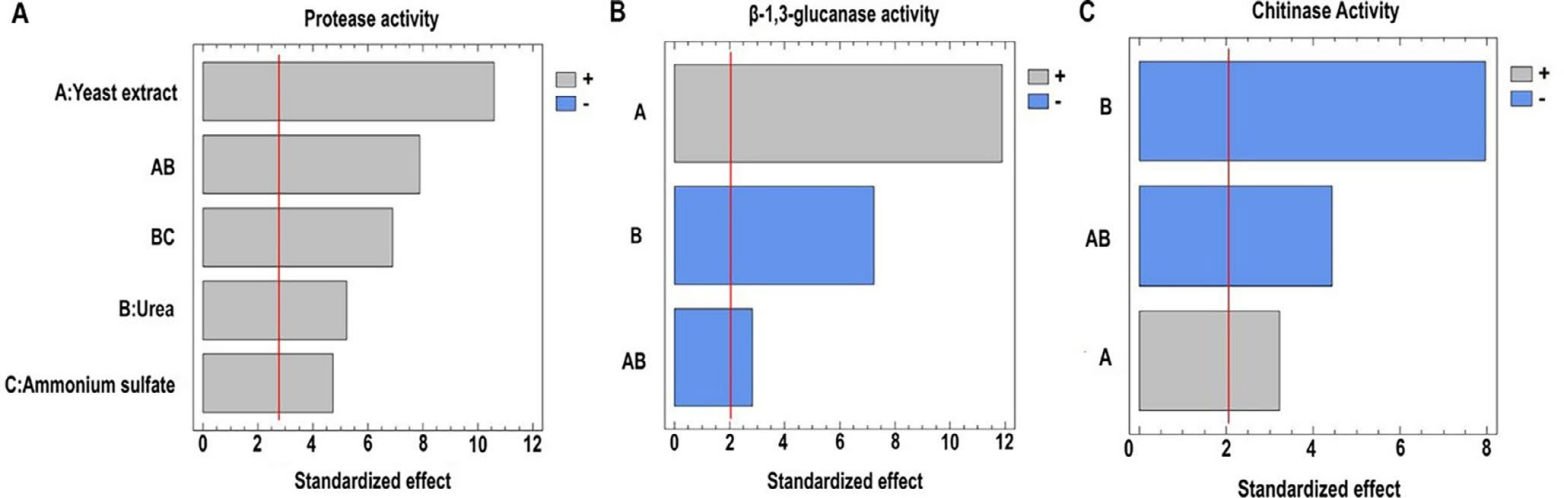
Pareto chart displaying factors in decreasing order of significance for (A) proteases, (B) β‐1,3‐glucanases and (C) chitinases of *Escovopsis primorosea* LBM 277. The bar length represents the standardised effect of each nitrogen source on enzyme activity, with a 95% confidence interval.

A statistically significant effect of the nitrogen sources, as well as the interaction between them (*p* < 0.05) (Table S1) The Pareto chart displays the effects on protease activity in order of decreasing significance. The three nitrogen sources and the interactions between urea–yeast extract and urea–ammonium sulphate showed a significant positive influence on protease activity (Figure 2A).

A mathematical model representing the relationship among the studied parameters, excluding non‐significant variables, was established with the following regression equation:

$$\begin{array}{c} \text{Protease}\;\left(U\;L^{-1}\right)=140.22+105.08\times A+51.8\times B\\ \;+47.08\times C+110.68\times \mathrm{AB}+96.81\times \mathrm{BC} \end{array}$$

Regarding β‐1,3‐glucanase activity, an *R* ^2^ of 90.78% was obtained and the lack‐of‐fit test was not significant (*p* > 0.05). Therefore, the analysis performed was appropriate for the data acquired. A significant influence of yeast extract, urea and their interaction (*p* < 0.05) was observed on β‐1,3‐glucanase activity (Table S2). Pareto chart demonstrated that yeast extract positively influenced the enzymatic activity (Figure 2B), while urea had a negative effect; that is, increased activity was obtained at its lower concentration, indicating a significant negative interaction between both variables.

Excluding non‐significant variables, the adjusted model equation was as follows:

$$\begin{array}{c} \beta -1,3-\text{glucanase}\;\left(U\;L^{-1}\right)=218.6+115.64\times A-70.46\\ \;\times B-86.08\times A^{2}-38.78\times \mathrm{AB} \end{array}$$

For chitinase activity, the *R* ^2^ value was 78.57% and the lack‐of‐fit test was not statistically significant (*p* > 0.05). The factors of yeast extract and urea and their interaction, significantly influenced enzymatic activity (*p* < 0.05) (Table S3). The Pareto chart showed that high urea levels negatively affected the activity, while high yeast extract levels positively influenced it. Therefore, the interaction between both factors was significantly negative (Figure 2C).

The adjusted model equation was as follows:

$$\begin{array}{c} \text{Chitinase}\;\left(U\;L^{-1}\right)=2.95+1.09\times A-1.84\times A^{2}-2.7\times B\\ \;-2.13\times \mathrm{AB}+1.6\times B^{2} \end{array}$$

**FIGURE 3:**
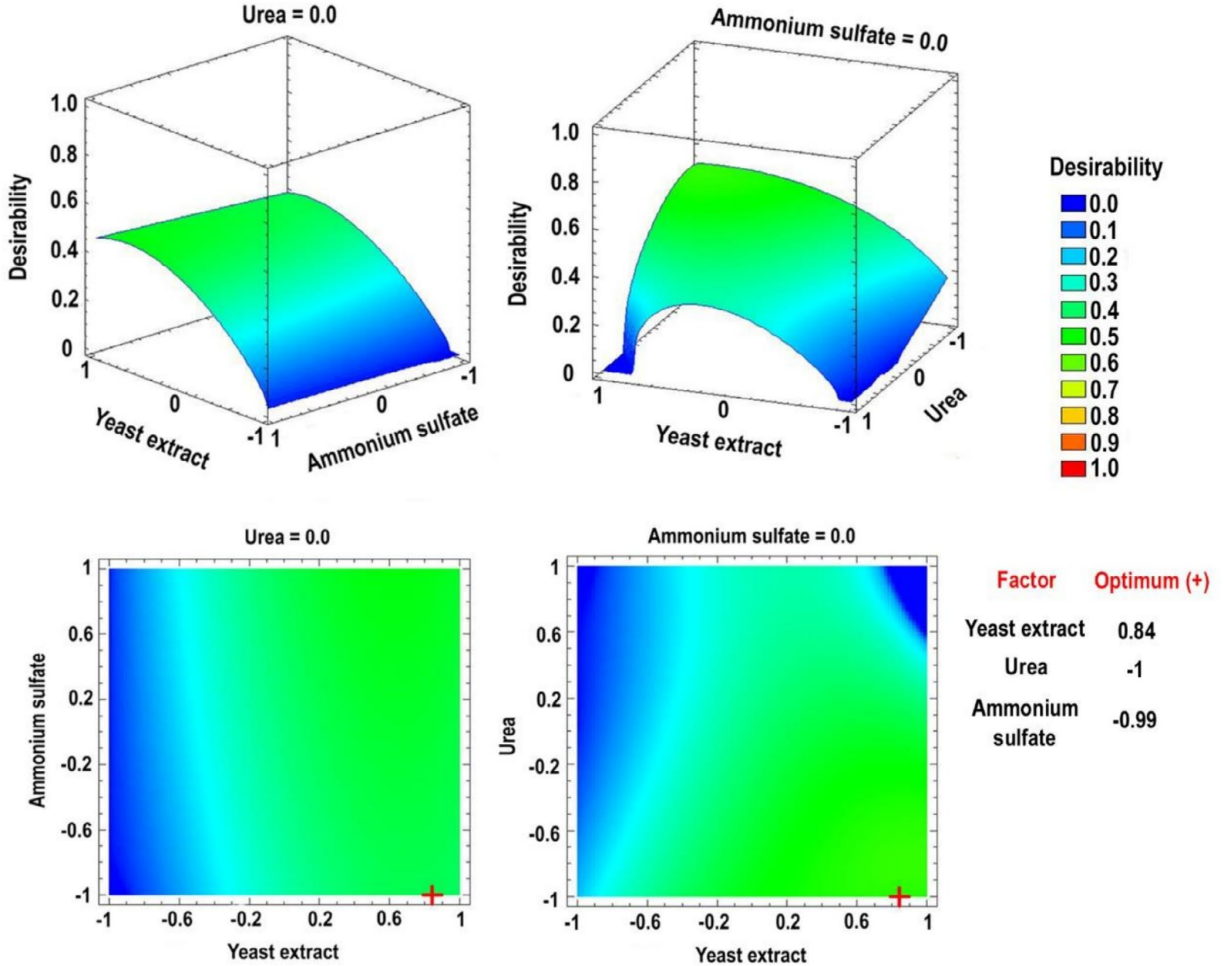
Estimated three‐dimensional response surface and response contour for nitrogen concentrations, with the optimal points (+) within the experimental design, to maximise desirability in *Escovopsis primorosea* LBM 277.

Given that the individual ANOVA‐RSM models predicted different optimal values for each enzymatic activity, a multiple‐response approach was applied. By constructing a desirability function from the models obtained for each enzyme, it was possible to determine the optimal combination of nitrogen sources that maximised the synergistic production of proteases, β‐1,3‐glucanases and chitinases. Three‐dimensional and contour RSM graphs were generated to visualise these optimal points within the experimental design (Figure 3).

Regarding the variable ammonium sulphate, the optimum was close to the low level. The yeast extract was located close to the high level and urea was found at the low level. From these results, the following optimal concentrations of each nitrogen component were calculated: urea (0.2 g L^−1^), yeast extract (1.86 g L^−1^) and ammonium sulphate (0.21 g L^−1^).

The predicted optimum of urea was located at one extreme of the design; therefore, two more concentrations were added in the following optimization assay.

#### 3.1.2 Initial pH, Inoculum Concentration and Urea Levels

**FIGURE 4:**
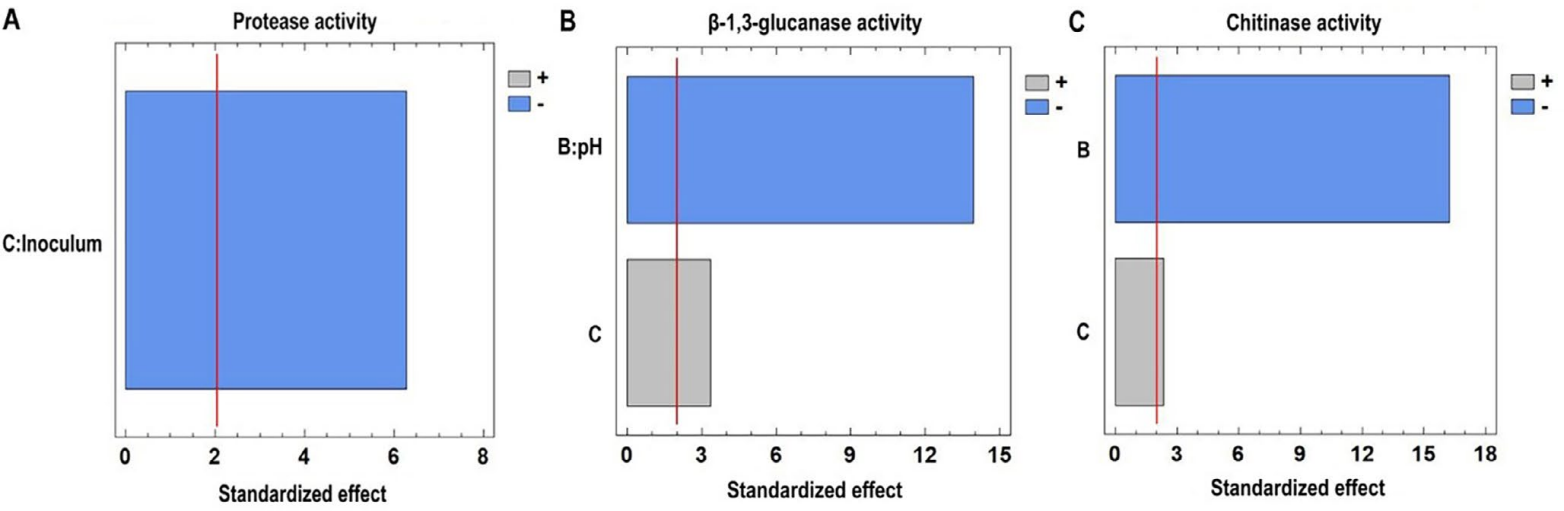
Pareto chart displaying factors (pH and inoculum concentration) in decreasing order of significance for (A) proteases, (B) β‐1,3‐glucanases and (C) chitinases of *Escovopsis primorosea* LBM 277. The bar length represents the standardised effect of each variable on enzyme activity, with a 95% confidence interval.

For protease activity, the *R* ^2^ value obtained in the RSM ANOVA was 92.34% and the lack‐of‐fit test was not significant (*p* > 0.05), confirming the adequacy of the selected model for describing the observed data (Table S4). Inoculum concentration had a statistically significant effect on enzymatic activity (*p* < 0.05), as indicated by the Pareto chart, which revealed a negative influence of this factor—higher enzymatic activity was observed at its lower level (−1) (Figure 4).

The regression equation of the fitted model, excluding non‐significant variables, was:

$$\begin{array}{c} \text{Protease}\;\left(U\;L^{-1}\right)=73.32-52.73\times C-37.03\\ \;\times A^{2}-24.19\times B^{2}+47.38\times C^{2} \end{array}$$

For β‐1,3‐glucanase activity, the *R* ^2^ value explained 87.00% of the observed variability and the lack‐of‐fit test was not significant (*p* > 0.05). Both pH and inoculum concentration significantly affected the enzymatic activity (Table S5). According to the Pareto chart, pH exhibited a negative effect at its highest level (+1), while inoculum concentration positively influenced enzymatic activity.

The regression equation of the fitted model, excluding non‐significant variables, was:

$$\begin{array}{c} \beta -1,3-\text{glucanase}\;\left(U\;L^{-1}\right)=549.58-240.83\times B+57.824\\ \;\times C-55.44\times A^{2}-188.45\times B^{2}-49.26\times C^{2} \end{array}$$

For chitinase activity, the *R* ^2^ value was 87.78% and the lack‐of‐fit test was not statistically significant (*p* > 0.05). The RSM ANOVA revealed that both pH and inoculum concentration significantly influenced the enzymatic activity (*p* < 0.05) (Table S6). The Pareto chart indicated that pH at its highest level (1) had a negative effect, whereas inoculum concentration positively influenced the enzymatic activity.

The regression equation of the fitted model, excluding non‐significant variables, was:

$$\begin{array}{c} \text{Chitinase}\;\left(U\;L^{-1}\right)=31.40-16.35\times B+2.36\\ \;\times C-11.62\times B^{2}-4.8\times C^{2} \end{array}$$

**FIGURE 5:**
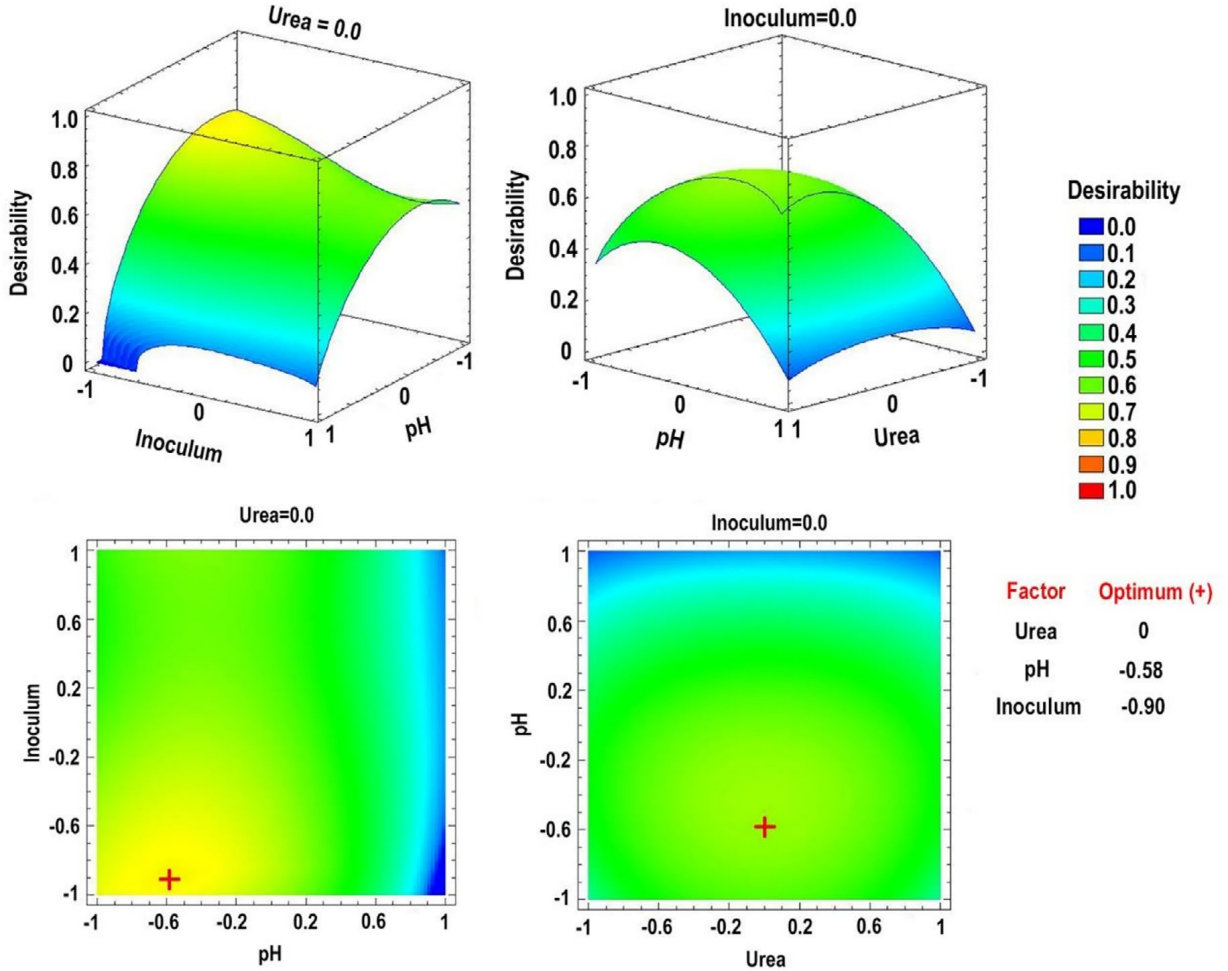
Estimated three‐dimensional response surface and contour plots with optimal points (+) within the experimental design, to optimise desirability for variables initial pH, inoculum concentration and urea levels in *Escovopsis primorosea* LBM 277.

Using multiple‐response optimization analysis, three‐dimensional RSM and contour plots were generated to determine the optimal conditions within the experimental region (Figure 5).

The optimum for urea concentration was found at the medium level. The optimal initial pH of the medium was close to the medium level, while the optimal inoculum concentration was near the lower level.

From these results, it was possible to predict the conditions that allow maximising the desirability within the experimental region, to optimise jointly the protease, β‐1,3‐glucanase and chitinase activities.

The optimal conditions, calculated from the predicted standard values, were: urea concentration (0.1 g L^−1^), pH (4.63) and inoculum concentration (4 × 10^6^ spores mL^−1^).

#### 3.1.3 Validation of the Optimised Experimental Variables

Experimental assays were conducted to validate the optimised conditions by comparing the predicted and actual values. The final enzymatic activity obtained was also compared with the initial pre‐optimization activity (Table 2).

**TABLE 2:** Experimental validation of the optimal conditions predicted for the activity of the CWDEs of *Escovopsis primorosea* LBM 277.

Enzyme	Pre‐optimisation activity (U L^−1^)	Predicted optimised activity (U L^−1^)	Experimental optimised activity (U L^−1^)
Proteases	23.6	152.3	83.8
β‐1,3‐glucanases	73.9	532.5	497.8
Chitinases	4.6	30.9	34.9

The experimental value obtained for protease activity was lower than predicted, indicating a lack of agreement. However, enzymatic activity showed a 71.4% increase (3.5‐fold) compared to the non‐optimised culture medium.

Regarding β‐1,3‐glucanase activity, the experimental value closely matched the predicted one (Table 2), achieving an 85.1% optimization (6.7‐fold increase) relative to the non‐optimised medium.

**FIGURE 6:**
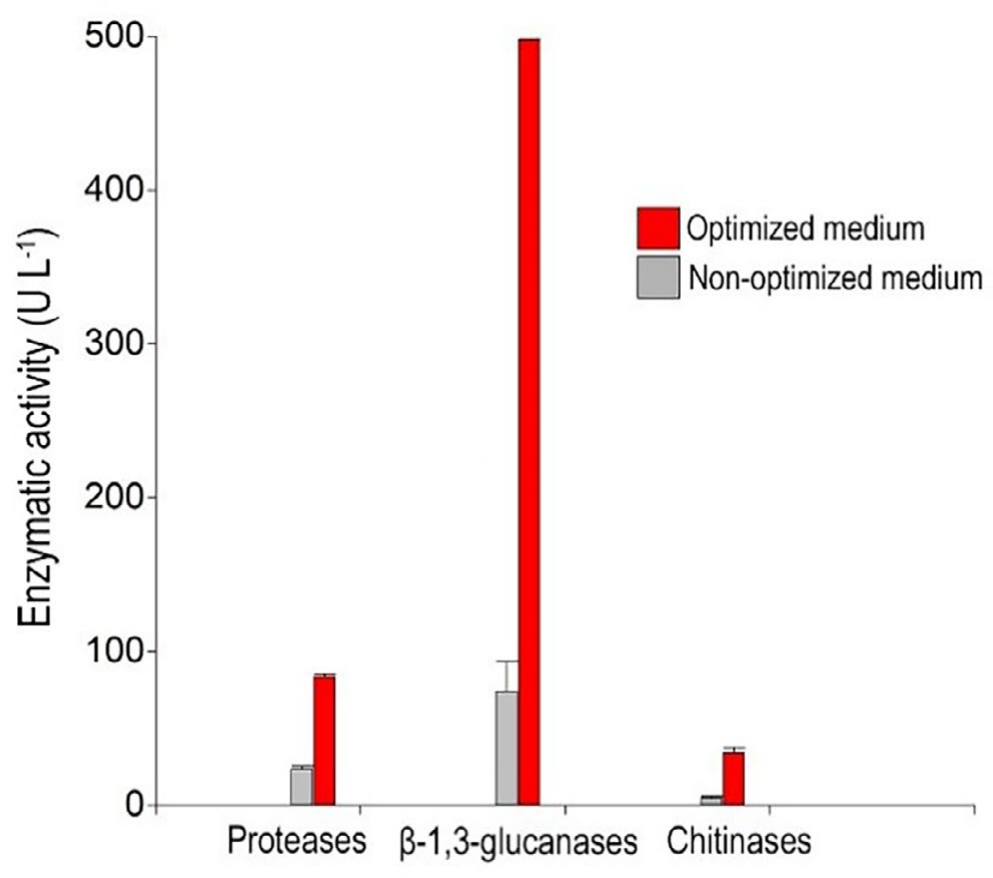
Total optimization of enzymatic activity for proteases, β‐1,3‐glucanases and chitinases in *Escovopsis primorosea* LBM 277.

Finally, the obtained value for chitinase activity aligned with the predicted one, demonstrating an 85.2% increase (6.8‐fold) compared to the non‐optimised medium (Figure 6).

### 3.2 Biochemical Characterisation of CWDEs Produced by *Escovopsis Primorosea* LBM 277 in the Optimised Supernatants

#### 3.2.1 Effect of Temperature and pH on CWDEs Activity

The effect of different reaction temperatures and pH values on the activity of CWDEs in the optimised supernatants was evaluated.

**FIGURE 7:**
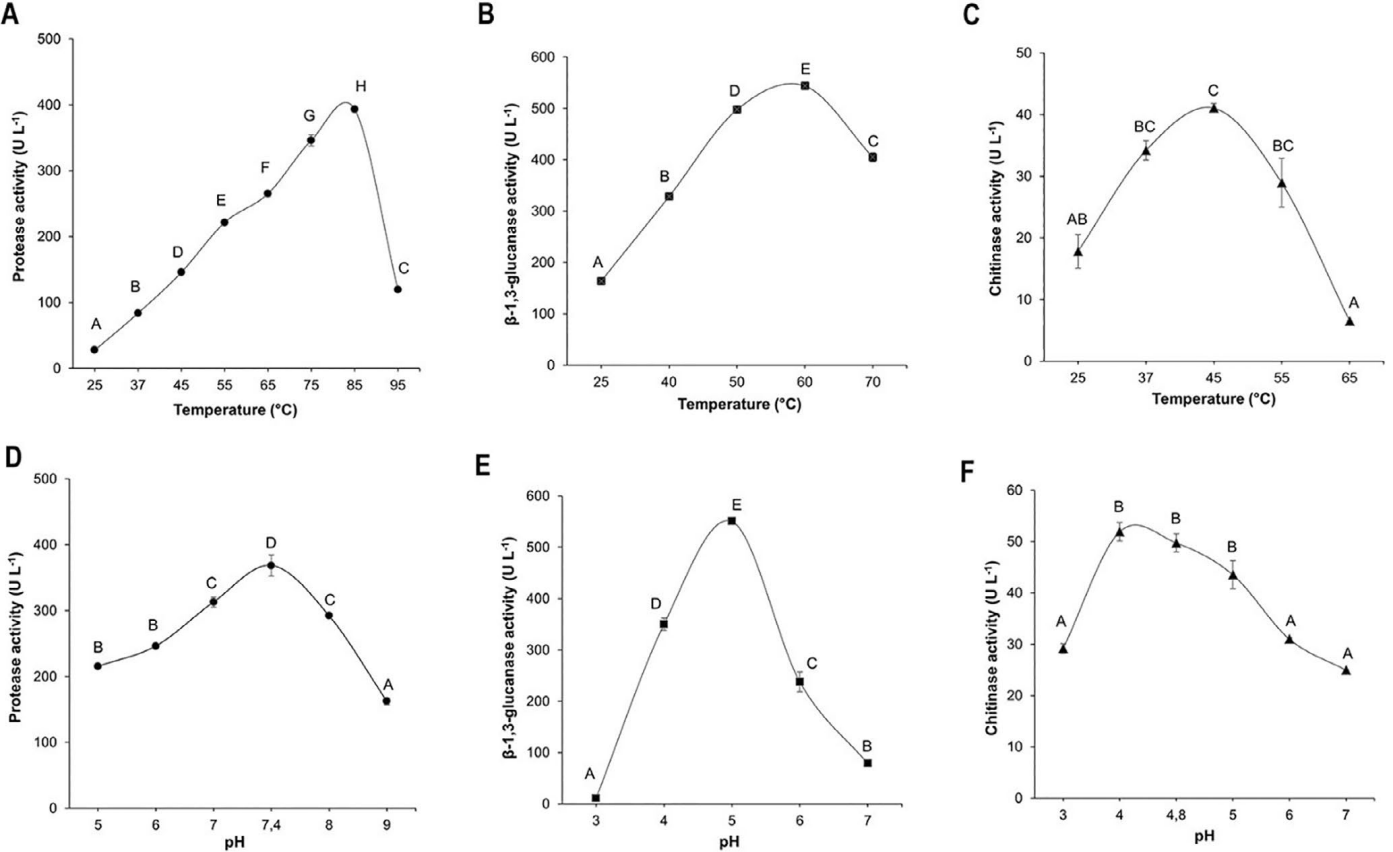
Effects of different reaction temperatures (A–C) and pH values (D–F) on the enzymatic activities of protease, β‐1,3‐glucanase and chitinase, respectively. Statistical differences were determined using Tukey's test at a 95% confidence level. Means followed by different letters indicate significant differences (*p* < 0.05).

For protease activity, maximum activity was observed at 85°C (393.15 ± 8.35 U L^−1^) and pH 7.4 (368.36 ± 31.72 U L^−1^) (Figure 7A,D), with a statistically significant difference (*p* < 0.05) compared to the other tested conditions.

For β‐1,3‐glucanases, the highest activity was recorded at 60°C (547.035 ± 6.69 U L^−1^) and pH 5 (550.36 ± 8.58 U L^−1^) (Figure 7B,E), showing a statistically significant difference (*p* < 0.05) relative to the other reaction conditions.

Concerning chitinases, no significant differences were observed in the temperature range of 37°C–55°C or between pH 4 and 5. However, the highest activity was detected at 45°C (41.074 ± 1.45 U L^−1^) and pH 4 (51.9 ± 2.54 U L^−1^) (Figure 7C,F).

#### 3.2.2 Thermal and pH‐Stability of CWDEs

**FIGURE 8:**
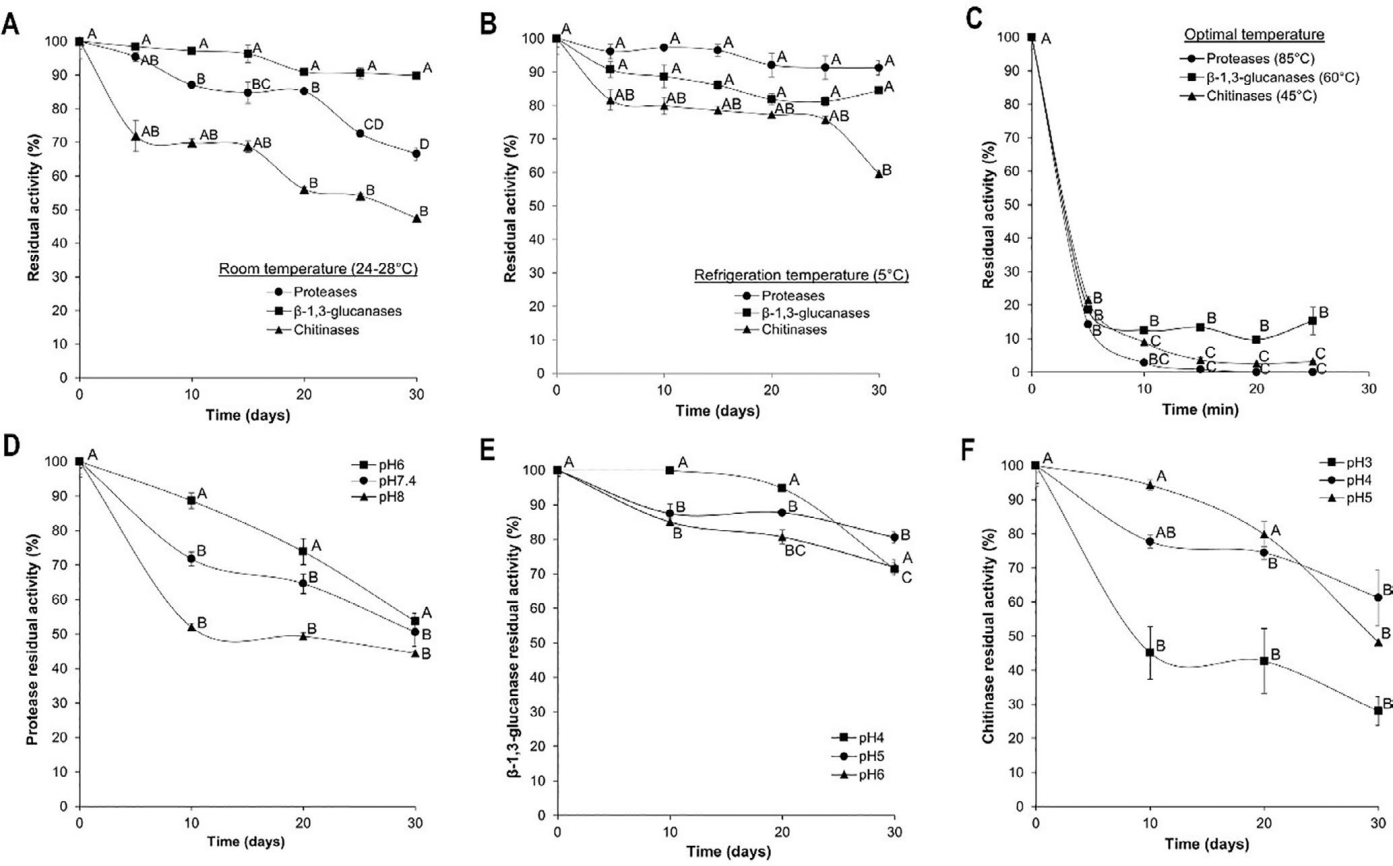
Thermostability and pH stability of protease, β‐1,3‐glucanase and chitinase activities from *Escovopsis primorosea* LBM 277. Residual enzyme activity over time under different incubation conditions: (A) refrigerated at 5°C, (B) at room temperature and (C) at the optimal reaction temperature for each enzyme. pH stability profiles over time for (D) proteases at pH 3, 4 and 5; (E) β‐1,3‐glucanases at pH 4, 5 and 6 and (F) chitinases at pH 3, 4 and 5. Statistical analysis was performed using Tukey's test at a 95% confidence level. Means with different letters indicate significant differences (*p* < 0.05).

Regarding thermal stability at refrigeration temperature (5°C), proteases and β‐1,3‐glucanases maintained stable activity throughout 30 days, with residual activity above 90% and 80%, respectively. On the other hand, chitinase activity remained above 70% for 25 days, without significant differences (*p* > 0.05) (Figure 8A).

At room temperature (24°C–28°C), residual protease activity showed a significant decline after 10 days (*p* < 0.05) but remained above 70% for 25 days. β‐1,3‐glucanase activity did not exhibit significant changes (*p* > 0.05) during the 30‐day incubation period, remaining above 90%. Chitinase activity remained above 70% for 15 days (Figure 8B).

Finally, at optimal reaction temperatures, the residual activity of all three enzymes decreased significantly (*p* < 0.05) after 5 min, dropping below 25% (Figure 8C).

Regarding pH stability at room temperature, the residual protease activity at pH 6 did not show significant differences. It remained above 70% for 20 days. At pH 7.4 and 8, a significant decline was detected by Day 10, though residual activity remained above 50% (Figure 8D).

For β‐1,3‐glucanases activity, at pH 4, no significant differences were observed. At pH 5 and 6, a significant decrease occurred by Day 10, but residual activity remained above 70% throughout the 30‐day incubation period (Figure 8E). For the chitinase activity at pH 3, a significant decrease occurred by day 10, with residual activity dropping below 50%. At pH 4 and 5, residual activity remained above 70% for 20 days (Figure 8F).

#### 3.2.3 Detection of Isoenzymes With CWDE Activity in ND‐PAGEs

Finally, zymograms were performed to selectively reveal the presence of isoenzymes by ND‐PAGE from the optimised supernatant of *E. primorosea* LBM 277.

**FIGURE 9:**
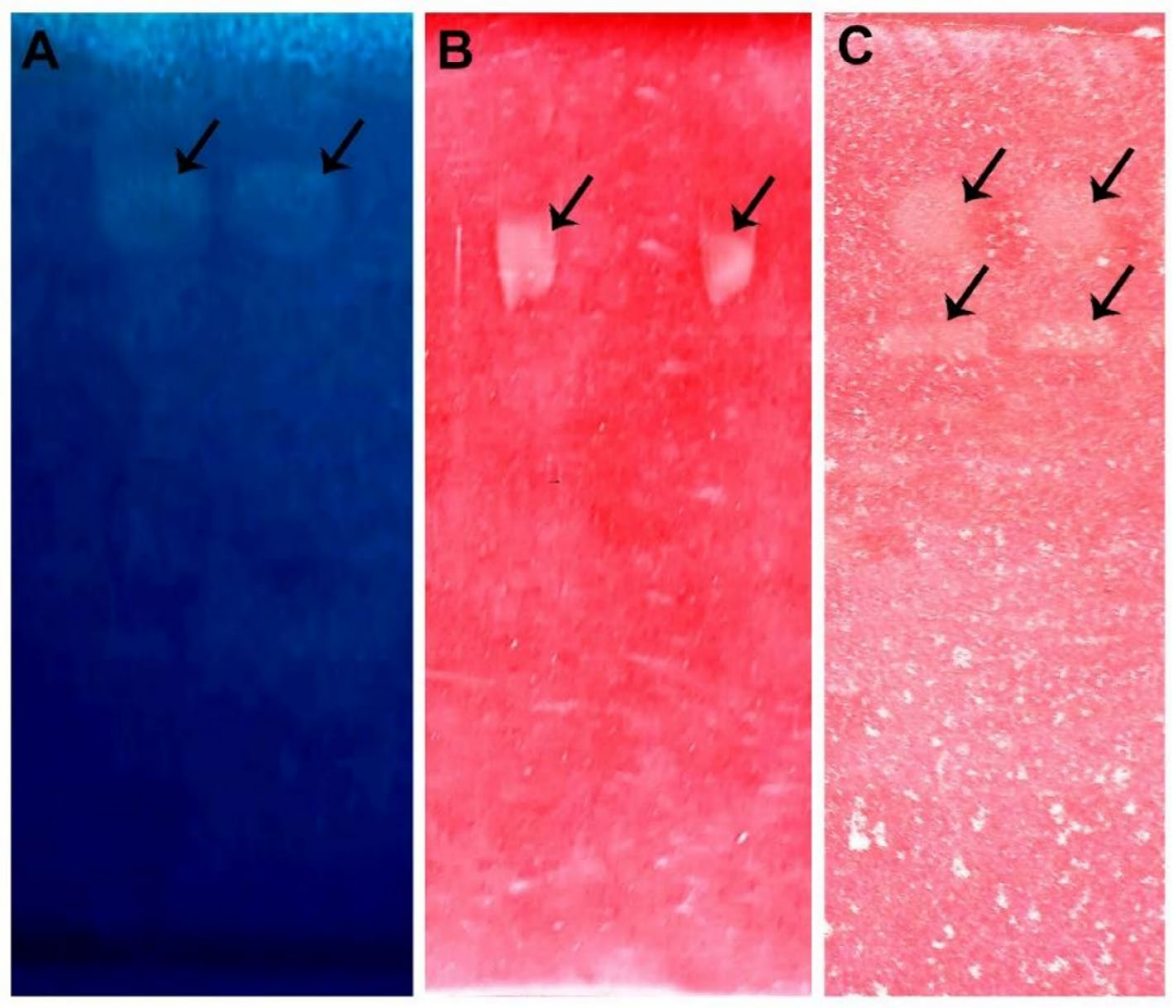
Detection of CWDEs from *Escovopsis primorosea* LBM 277. ND‐PAGE analysis of optimised supernatants, loaded in duplicate, showing isoenzyme profiles of proteases (A) (Coomassie Brilliant Blue staining), β‐1,3‐glucanases (B) and chitinases (C) (Congo red staining). Arrows indicate zones of enzymatic activity.

Different substrates were used and it was possible to perform the isoenzyme profile of the three CWDEs (Figure 9). A single band was detected for proteases and β‐1,3‐glucanases, while two bands were observed for chitinases, indicating the presence of two isoenzymes for this enzyme.

These results indicate that *E. primorosea* LBM 277, under inductive fermentation conditions, produces a CWDEs system consisting of at least one protease enzyme, one β‐1,3‐glucanase and two chitinases. This enzymatic induction is due to the incorporation of fungal cell walls of *L. gongylophorus* as a carbon source and the Mandels nitrogen complex as a nitrogen source.

## 4 Discussion

The findings of this study demonstrate that *E. primorosea* LBM 277 produces a robust CWDEs system, composed primarily of proteases, β‐1,3‐glucanases and chitinases when cultivated with *L. gongylophorus* cell walls as the primary carbon source.

The experimental designs allowed for an efficient analysis of the correlation between the components of the culture medium and the secretion of enzymes of biotechnological interest.

The extracellular production of CWDEs by *Escovopsis* has been scarcely explored. Notably, this is the first study to quantitatively determine the secretion of β‐1,3‐glucanases by a strain of *Escovopsis*. Previously, our team established the reaction conditions to quantify protease activity in *Escovopsis* supernatants (Barengo et al. 2020). Additionally, Inglis and Kawchuk (2002) documented the secretion of chitinases by *Escovopsis*.

There are reports of an increased secretion of proteases, chitinases and β‐1,3‐glucanases when mycoparasitic fungi are cultivated in media containing fungal cell walls as a carbon source (Markovich and Kononova 2003; Seidl 2008; Ye et al. 2023). This response is attributed to the synergistic interaction of CWDEs, which are induced by complex substrates until they are degraded into simpler components that the microorganism can utilise for growth (Inglis and Kawchuk 2002; Viterbo et al. 2002). Based on this premise, *L. gongylophorus* cell walls were used in this study to stimulate the production of the CWDEs complex in *E. primorosea* LBM 277.

In addition to the role of fungal cell walls as inducers of CWDEs production, substrate concentration is a key factor in microbial fermentation (Singh et al. 2007; Adetiloye et al. 2025). This study showed that the highest activities of proteases, β‐1,3‐glucanases and chitinases were observed at one of the lowest concentrations of the *L. gongylophorus* cell walls assayed.

This observation is consistent with previous reports on filamentous fungi, where increased enzymatic activity has been associated with the induction of cell walls at limiting concentrations (Gupta et al. 1993; Haran et al. 1996; Abidi et al. 2007). This effect may result from mycelial autolysis or the degradation of cell wall components, which are subsequently excreted into the culture medium and utilised as alternative carbon sources when easily metabolizable substrates are scarce (de la Cruz Díaz 1994).

Nitrogen availability is another crucial factor influencing enzyme production. Several studies highlight the favourable impact of Mandels medium on enzyme secretion due to its balanced combination of organic and inorganic nitrogen sources, which play a key role in metabolic regulation, including enzyme expression (Oberoi et al. 2014; Vijay Kalaskar et al. 2014).

In this study, an RSM‐BBD design was applied to optimise nitrogen sources. This methodology was selected, as it enables the simultaneous evaluation of multiple variables and their interactions with a reduced number of experiments compared to one‐factor‐at‐a‐time approaches. This design has been successfully applied in microbial enzyme optimization, providing statistically reliable models for prediction and process improvement (Wasli et al. 2009; Dukariya and Kumar 2021; Gares et al. 2023). Therefore, this statistical approach enabled the development of correlation models for CWDEs produced by *E. primorosea* LBM 277.

A significant positive influence was observed on the three nitrogen sources on protease activity. Organic compounds such as urea and yeast extract have been reported to enhance protease production in several microorganisms (Sharma 2019). Likewise, other studies indicate that protease performance improves when inorganic sources, such as ammonium sulphate, are used (do Nascimento and Martins 2004; Pawar et al. 2023).

Yeast extract also positively influenced β‐1,3‐glucanase and chitinase activities, consistent with previous reports indicating that its incorporation into the culture medium stimulates the secretion of these enzymes (Alamri et al. 2016; Mezadri et al. 2022). In contrast, urea displayed a negative influence, producing an antagonistic interaction with yeast extract. This finding supports previous studies showing that excessive urea supplementation can reduce enzymatic performance (Sandhya et al. 2004; Rao et al. 2016).

In the next optimization step, the optimal concentration was determined between the absence of urea and the lower limit predicted in the previous design. These findings suggest that their complete absence also reduces enzymatic production.

On the other hand, lower pH values significantly favoured the production of β‐1,3‐glucanase and chitinase. Other studies have reported that acidic conditions are crucial for the optimal activity of these enzymes (Nampoothiri et al. 2004; Moreno‐Mateos et al. 2007). These enzymes exhibited coordinated behaviour, aligning with findings by Rao et al. (2016), who suggested that their secretion may be co‐regulated.

Inoculum concentration had a significant inhibitory effect on the production of all three CWDEs. This may be attributed to the rapid proliferation and biomass accumulation facilitated by a high initial spore concentration. Nutrient competition likely suppresses metabolic activity, reducing enzyme secretion beyond a certain threshold (Nampoothiri et al. 2004).

After experimental validation, the activities of β‐1,3‐glucanase and chitinase were found to be in close agreement with the model predictions. In contrast, the experimental protease activity deviated from the predicted value, reflecting its distinct response to the optimization variables.

The optimization results were promising for the CWDEs production of *E. primorosea* LBM 277. Notably, this is the first study to apply RSM and to optimise multiple responses to the *Escovopsis* genus.

Following the optimization of culture conditions, the biochemical characterisation of the CWDEs secreted by *E. primorosea* LBM 277 is crucial to understanding their potential biological role and applicability (Haard and Simpson 2000).

The optimised supernatants presented detectable CWDEs activity across the tested temperature and pH ranges. Their functionality at ambient temperatures is particularly relevant, as the internal microclimate of leaf‐cutter ant nests, regulated by the metabolism of *L. gongylophorus*, remains relatively stable near room temperature (Quirán and Pilati 1998).

Proteases exhibited an optimal reaction temperature that is higher than the commonly reported range of 30°C–60°C for fungal proteases (Pawar et al. 2023), indicating that these enzymes display thermotolerant behaviour. Similar values have been observed in bacterial studies of alkaline proteases from *Bacillus* spp., where optimal temperatures around 85°C have been documented (Sookkheo et al. 2000; Nilegaonkar et al. 2008). The optimal pH was consistent with reported values for fungal alkaline proteases (Jayalakshmi et al. 2009; Aissaoui et al. 2017). This class of enzymes, primarily composed of serine proteases, has been strongly associated with mycoparasitism and biocontrol processes, as observed in *Trichoderma* spp. (Pozo et al. 2004; Rao et al. 2016).

Regarding β‐1,3‐glucanases, studies have reported optimal temperatures for fungal enzymes ranging from 50°C to 65°C (Marco and de Marco and Felix 2007; O'Connell et al. 2011; Periyasamy et al. 2017). Regarding pH, the activity of β‐1,3‐glucanases generally falls within the 4–6 range (Pitson et al. 1995; Periyasamy et al. 2017).

Chitinases exhibited an optimal temperature range consistent with previous reports that indicate broad temperature optima (23°C–55°C) for fungal chitinases (Loc et al. 2020; Chung et al. 2022). Additionally, chitinase activity peaked at pH values between 4 and 5, aligning with optimal pH values reported for *Trichoderma* strains (Thakur et al. 2020).

On the other hand, the stability of CWDEs is a critical factor in most bioprocesses, as it directly influences the application efficacy, production stage, recovery and purification (Illanes 1999; Carrera 2003). Ensuring enzyme stability is particularly important for their application in biological control strategies.

In this study, the CWDEs exhibited remarkable stability under refrigeration and at room temperature. These are key characteristics, as they would facilitate the conservation of a bioproduct based on the enzymes of *E. primorosea* LBM 277 but also ensure that the enzyme supernatants maintain high stability for at least 30 days. This suggests the potential for a long‐lasting biocontrol effect.

In contrast, at optimal reaction temperatures, the enzymatic stability was significantly reduced in minutes. This rapid decline is attributed to heat denaturation and autodigestion of the enzymes in the crude supernatant (El‐Katatny 2008).

It is worth noting that, despite the high optimum temperature observed for protease activity, thermal stability assays showed that the proteases are thermotolerant but not thermostable.

On the other hand, the supernatants exhibited stability across a broad pH range. Regarding this point, reports on the impact of leaf‐cutter ants on soil properties indicate that nest environments typically range from slightly acidic to slightly alkaline, often tending towards neutrality compared to surrounding soils (Frouz and Jilková 2008; Sabattini et al. 2020). However, fungi acidify their microenvironment to maintain optimal physiological conditions (Joergensen and Wichern 2008; Rousk et al. 2009). Therefore, the internal pH of the nest's fungal garden is likely slightly acidic. In this context, the pH stability of *E. primorosea* LBM 277 CWDEs aligns well with the conditions required for their potential application in leaf‐cutter ant nests.

Finally, the diversity of enzymes with the same activity has been interpreted as a molecular strategy for optimising substrate degradation, allowing for a more efficient breakdown of the targeted components (Uhlig 1998; Micales and Bonde 2017).

In this study, zymography was used to determine the presence of isozymes secreted by *E. primorosea* LBM 277 under optimised fermentation conditions. For proteases and β‐1,3‐glucanases, a single enzyme was observed for each one. Notably, this is the first report to characterise these *Escovopsis* enzymes using polyacrylamide gel electrophoresis.

In contrast, two isoforms of chitinases were detected, in agreement with the only previous report by Inglis and Kawchuk (2002), who cultivated *Escovopsis* sp. with *Rhizoctonia solani* cell walls as the sole carbon source.

Further genomic evidence supports the enzymatic repertoire of this genus. de Man et al. (2016) identified genes encoding chitinases (GH18, GH20) and β‐1,3‐glucanases (GH16) in the genome of *Escovopsis weberi* . More recently, Gotting et al. (2022), in a genomic and metabolomic analysis of the genus *Escovopsis*, reported proteases potentially involved in fungal virulence, along with several predicted enzymes associated with chitin degradation, including GH16, GH18, Q8J1Y3_BEABA, O59928_HYPVI, AA11 and AA7. The authors emphasised the evolutionary conservation of chitinolytic genes within the core secretome of these highly specialised fungus‐garden mycoparasites.

These findings indicate that *E. primorosea* LBM 277, under inductive culture conditions, produces a CWDEs complex composed of at least one protease, one β‐1,3‐glucanase and two chitinases. This enzymatic repertoire highlights the potential of *E. primorosea* LBM 277 as a source of hydrolytic enzymes for biotechnological applications.

From an applied perspective, this enzymatic system offers an eco‐friendly alternative to harness the natural antagonism of the ant–symbiont fungus–parasite interaction, in which Escovopsis species are specialised mycoparasites of *L. gongylophorus* (Currie et al. 2003). The controlled use of enzymatic preparations will allow a targeted attack on the structural components *of L. gongylophorus* cell walls, while preventing potential impacts on non‐target soil fungi or other members of the ant‐associated microbiota. Such an approach aligns with the principles of sustainable biological control, emphasising efficacy, selectivity and biosafety (Singh et al. 2022; Tyagi et al. 2024). Posterior studies will therefore evaluate enzyme persistence, immobilised strategies, ecological compatibility and field performance under realistic agroecosystem conditions.

## 5 Conclusions

This work demonstrates the capacity of the mycoparasitic fungus *E. primorosea* LBM277 to produce CWDEs efficiently under SmF. By applying factorial and RSM–BBD experimental designs, we identified critical nutritional and physicochemical parameters that govern the coordinated secretion of proteases, β‐1,3‐glucanases and chitinases. The optimised enzymatic complex showed remarkable stability under conditions consistent with the physicochemical microenvironment of leaf‐cutter ant fungal gardens.

Beyond methodological optimization, these findings enhance our understanding of *Escovopsis* physiology and underscore its potential as a biotechnological resource for developing enzyme‐based strategies targeting the mutualistic fungus *L. gongylophorus*. Such an indirect biocontrol approach, based on eco‐compatible enzymatic formulations, could contribute to the sustainable management of leaf‐cutter ants, a significant pest in agroforestry systems.

## Author Contributions

**Gustavo Ángel Bich:** writing – review and editing, project administration. **María Lorena Castrillo:** conceptualization, writing – review and editing, project administration, supervision. **Pedro Darío Zapata:** writing – review and editing, supervision. **Marcela Paola Barengo:** conceptualization, investigation, writing – original draft, methodology, validation, formal analysis. **Natalia Soledad Amerio:** conceptualization, investigation, methodology.

## Funding

This work was supported by the Agencia Nacional de Promoción Científica y Tecnológica (PICT 2016‐3349, PICT 2018‐04348, PICT 2021 GRF TI 00528 and PIP 2022‐2024 GI 11220210100916CO01) and Consejo Nacional de Investigaciones Científicas y Técnicas.

## Conflicts of Interest

The authors declare no conflicts of interest.

## Supporting information


**Table S1:** ANOVA of RSM of nitrogen sources, for protease activity in *E. primorosea* LBM 277 (95.0% confidence level).
**Table S2:** ANOVA of RSM of nitrogen sources, for β‐1,3‐glucanase activity in *Escovopsis primorosea* LBM 277 (95.0% confidence level).
**Table S3:** ANOVA of RSM of nitrogen sources, for chitinase activity in *Escovopsis primorosea* LBM 277 (95.0% confidence level).
**Table S4:** ANOVA of RSM of Initial pH, inoculum concentration and urea levels, for protease activity in *Escovopsis primorosea* LBM 277 (95.0% confidence level).
**Table S5:** ANOVA of RSM of Initial pH, inoculum concentration and urea levels, for β‐1,3‐glucanase activity in *Escovopsis primorosea* LBM 277 (95.0% confidence level).
**Table S6:** ANOVA of RSM of Initial pH, inoculum concentration and urea levels, for chitinase activity in *Escovopsis primorosea* LBM 277 (95.0% confidence level).

## Data Availability

The data that support the findings of this study are available in the Supporting Information and from the corresponding author upon reasonable request.
