# Supplementary material for: Towards a Novel Biocontrol Strategy: High Performance of Optimised Cell Wall‐Degrading Enzymes Secreted by Escovopsis primorosea LBM 277
Source: Environ Microbiol Rep. 2026 Mar 18;18(2):e70271. doi: 10.1111/1758-2229.70271 (PMC13140752; doi:10.1111/1758-2229.70271)
Supplement: Supplementary file 1 — Table S1: ANOVA of RSM of nitrogen sources, for protease activity in E. primorosea LBM 277 (95.0% confidence level). Table S2: ANOVA of RSM of nitrogen sources, for β‐1,3‐glucanase activity in Escovopsis primorosea LBM 277 (95.0% confidence level). Table S3: ANOVA of RSM of nitrogen sources, for chitinase activity in Escovopsis primorosea LBM 277 (95.0% confidence level). Table S4: ANOVA of RSM of Initial pH, inoculum concentration and urea levels, for protease activity in Escovopsis primorosea LBM 277 (95.0% confidence level). Table S5: ANOVA of RSM of Initial pH, inoculum concentration and urea levels, for β‐1,3‐glucanase activity in Escovopsis primorosea LBM 277 (95.0% confidence level). Table S6: ANOVA of RSM of Initial pH, inoculum concentration and urea levels, for chitinase activity in Escovopsis primorosea LBM 277 (95.0% confidence level). [file EMI4-18-e70271-s001.docx]

Supplementary Information to:

**Toward a Novel Biocontrol Strategy: High performance of optimized cell wall-degrading enzymes secreted by *Escovopsis primorosea* LBM 277**

Marcela Paola Barengo^1.2^. Natalia Soledad Amerio^1.2^. Gustavo Ángel Bich^1.2^. Pedro Darío Zapata^1.2^. María Lorena Castrillo^1.2^

1. Molecular Biotechnology Laboratory. Institute of Biotechnology Misiones “Dra. María Ebe Reca”. Faculty of Exact Chemical and Natural Sciences. National University of Misiones. Argentina.

2. CONICET (National Scientific and Technical Research Council). Buenos Aires. Argentina.

This supplementary information contains Supplementary Tables S1-S6

**Table S1. ANOVA of RSM of nitrogen sources, for protease activity in *E. primorosea* LBM 277 (95.0% confidence level).**

| *Source* | *Sum of Squares* | *DF* | *Mean Square* | *F-Ratio* | *P-Value* |
| --- | --- | --- | --- | --- | --- |
| A: Yeast extract | 88328.8 | 1 | 88328.8 | 111.96 | 0.0005 |
| B: Urea | 21466.2 | 1 | 21466.2 | 27.21 | 0.0064 |
| C: Ammonium sulfate | 17730.7 | 1 | 17730.7 | 22.47 | 0.0090 |
| AB | 49004.5 | 1 | 49004.5 | 62.11 | 0.0014 |
| BC | 37490.6 | 1 | 37490.6 | 47.52 | 0.0023 |
| Lack-of-fit | 28656.7 | 7 | 4093.81 | 5.19 | 0.0654 |
| Pure error | 3155.8 | 4 | 788.949 |  |  |
| Total (corr.) | 245833. | 16 |  |  |  |

**Table S2. ANOVA of RSM of nitrogen sources, for β-1,3-glucanase activity in *E. primorosea* LBM 277 (95.0% confidence level).**

| *Source* | *Sum of Squares* | *DF* | *Mean Square* | *F-Ratio* | *P-Value* |
| --- | --- | --- | --- | --- | --- |
| A: Yeast extract | 213955. | 1 | 213955. | 141.04 | 0.0000 |
| B: Urea | 79444.9 | 1 | 79444.9 | 52.37 | 0.0000 |
| AA | 62763.3 | 1 | 62763.3 | 41.37 | 0.0000 |
| AB | *12031.7* | 1 | 12031.7 | 7.93 | 0.0093 |
| Lack-of-fit | 4367.55 | 4 | 1091.89 | 0.72 | 0.5865 |
| Pure error | 37925.3 | 25 | 1517.01 |  |  |
| Total (corr.) | 410488. | 33 |  |  |  |

**Table S3. ANOVA of RSM of nitrogen sources, for chitinase activity in *E. primorosea* LBM 277 (95.0% confidence level).**

| *Source* | *Sum of Squares* | *DF* | *Mean Square* | *F-Ratio* | *P-Value* |
| --- | --- | --- | --- | --- | --- |
| A: Yeast extract | 19.0991 | 1 | 19.0991 | 10.40 | 0.0035 |
| B: Urea | 116.602 | 1 | 116.602 | 63.51 | 0.0000 |
| AA | 28.7724 | 1 | 28.7724 | 15.67 | 0.0006 |
| AB | 36.1718 | 1 | 36.1718 | 19.70 | 0.0002 |
| BB | 21.7091 | 1 | 21.7091 | 11.82 | 0.0021 |
| Lack-of-fit | 14.0059 | 3 | 4.66862 | 2.54 | 0.0790 |
| Pure error | 45.8983 | 25 | 1.83593 |  |  |
| Total (corr.) | 279.629 | 33 |  |  |  |

**Table S4. ANOVA of RSM of Initial pH, Inoculum Concentration, and Urea Levels, for protease activity in *E. primorosea* LBM 277 (95.0% confidence level).**

| *Source* | *Sum of Squares* | *DF* | *Mean Square* | *F-Ratio* | *P-Value* |
| --- | --- | --- | --- | --- | --- |
| C: Inoculum | 33369.7 | 1 | 33369.7 | 140.45 | 0.0000 |
| AA | 9139.81 | 1 | 9139.81 | 38.47 | 0.0000 |
| BB | 3900.76 | 1 | 3900.76 | 16.42 | 0.0005 |
| CC | 14970.3 | 1 | 14970.3 | 63.01 | 0.0000 |
| Lack-of-fit | 97.6067 | 1 | 97.6067 | 0.41 | 0.5276 |
| Pure error | 5702.11 | 24 | 237.588 |  |  |
| Total (corr.) | 68872.0 | 29 |  |  |  |

**Table S5. ANOVA of RSM of Initial pH, Inoculum Concentration, and Urea Levels, for β-1,3-glucanase activity in *E. primorosea* LBM 277 (95.0% confidence level).**

| *Source* | *Sum of Squares* | *DF* | *Mean Square* | *F-Ratio* | *P-Value* |
| --- | --- | --- | --- | --- | --- |
| B: pH | 1.39192E6 | 1 | 1.39192E6 | 194.84 | 0.0000 |
| C: Inoculum | 80246.1 | 1 | 80246.1 | 11.23 | 0.0017 |
| AA | 38822.5 | 1 | 38822.5 | 5.43 | 0.0246 |
| BB | 448588. | 1 | 448588. | 62.79 | 0.0000 |
| CC | 30655.7 | 1 | 30655.7 | 4.29 | 0.0445 |
| Lack-of-fit | 59331.5 | 3 | 19777.2 | 2.77 | 0.0534 |
| Pure error | 300039. | 42 | 7143.79 |  |  |
| Total (corr.) | 2.38436E6 | 50 |  |  |  |

**Table S6. ANOVA of RSM of Initial pH, Inoculum Concentration, and Urea Levels, for chitinase activity in *E. primorosea* LBM 277 (95.0% confidence level).**

| *Source* | *Sum of Squares* | *DF* | *Mean Square* | *F-Ratio* | *P-Value* |
| --- | --- | --- | --- | --- | --- |
| B: pH | 6417.05 | 1 | 6417.05 | 264.34 | 0.0000 |
| C: Inoculum | 134.237 | 1 | 134.237 | 5.53 | 0.0235 |
| BB | 1710.15 | 1 | 1710.15 | 70.45 | 0.0000 |
| CC | 291.979 | 1 | 291.979 | 12.03 | 0.0012 |
| Lack-of-fit | 183.122 | 4 | 45.7806 | 1.89 | 0.1308 |
| Pure error | 1019.57 | 42 | 24.2755 |  |  |
| Total (corr.) | 9841.07 | 50 |  |  |  |
